# A Rare Case of Recurrent Immunotactoid Glomerulonephritis

**DOI:** 10.7759/cureus.35136

**Published:** 2023-02-18

**Authors:** Mohankumar Doraiswamy, Samir V Parikh, Sergey Brodsky

**Affiliations:** 1 Department of Internal Medicine, Arkansas College of Osteopathic Medicine, Fort Smith, USA; 2 Depatment of Internal Medicine, Mercy Health, Fort Smith, USA; 3 Department of Internal Medicine, The Ohio State University Wexner Medical Center, Columbus, USA; 4 Department of Pathology, The Ohio State University Wexner Medical Center, Columbus, USA

**Keywords:** nephrology disorders, general nephrology dialysis and transplanation, internal medicine. clinical nephrology, general nephrology, nephrology, glomerular disease, immunosuppression, kidney biopsy, glomerulonephritis, immunotactoid

## Abstract

Immunotactoid glomerulopathy is an uncommon cause of glomerular disease that results from deposits derived from immunoglobulins. This rare disease can occur in native kidneys and in transplant patients. They are present only in 0.5% to 1.4% of native kidney biopsies. Treatment of this disease is directed at the underlying monoclonal gammopathy, infection, and B-cell lymphoproliferative disorders. Prognosis is very guarded with 50% of people developing ESRD within five years of diagnosis. We present an interesting, rare case of recurrent immunotactoid glomerulonephritis which responded appropriately to rituximab therapy.

## Introduction

Organized fibrillar or microtubular glomerular deposits may be encountered in a variety of renal disorders, including amyloidosis, cryoglobulinemic glomerulonephritis (GN), lupus nephritis, collagen glomerulopathies, and the entities of fibrillary and Immunotactoid GN [1]. Immunotactoid glomerulopathy [ITG] is an uncommon, but distinctly different, cause of glomerular disease that results from deposits derived from immunoglobulins [2]. ITG is associated with larger microtubular deposits, usually >30 nm in diameter, which is often hollow and arranged in parallel or stacked arrays. ITG is differentiated from fibrillary GN (FG) by the identification of underlying paraproteinemia or autoimmune disorders where most of the latter patients have no underlying systemic disorder. ITG is present in 0.5% to 1.4% of native kidney biopsies [3]. The identification of the protein DnaJ heat shock protein (DNAJB9) in the glomeruli of patients differentiates fibrillary GN from immunotactoid GN [4]. The rates of recurrence in ITG have been not well studied. A study from Australia and New Zealand dialysis and transplant registry reported the prognosis of 11 patients with ITG [5]. Patients with ITG had inferior survival on dialysis compared with those patients with other causes of end-stage renal disease (ESRD). Among patients who are transplanted, the recurrent disease was seen in one of four patients with ITG. We presented an interesting case of recurrent ITG after achieving remission for one year of initial presentation who responded very well to treatment without necessitating the need for dialysis.

## Case presentation

**Figure 1:**
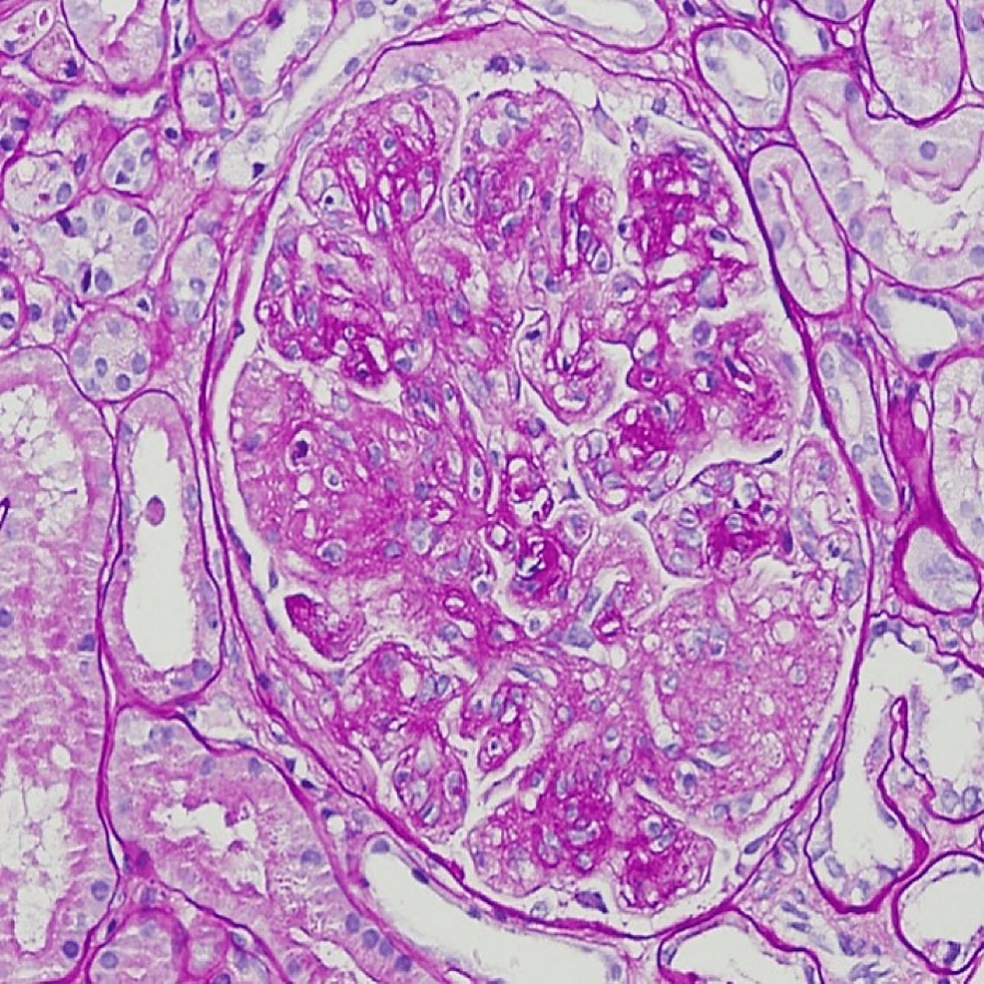
Light microscopy, periodic Acid-Schiff stain (PAS) shows membranoproliferative (thick capillary walls with increased cellularity) pattern of glomerulonephritis

**Figure 2:**
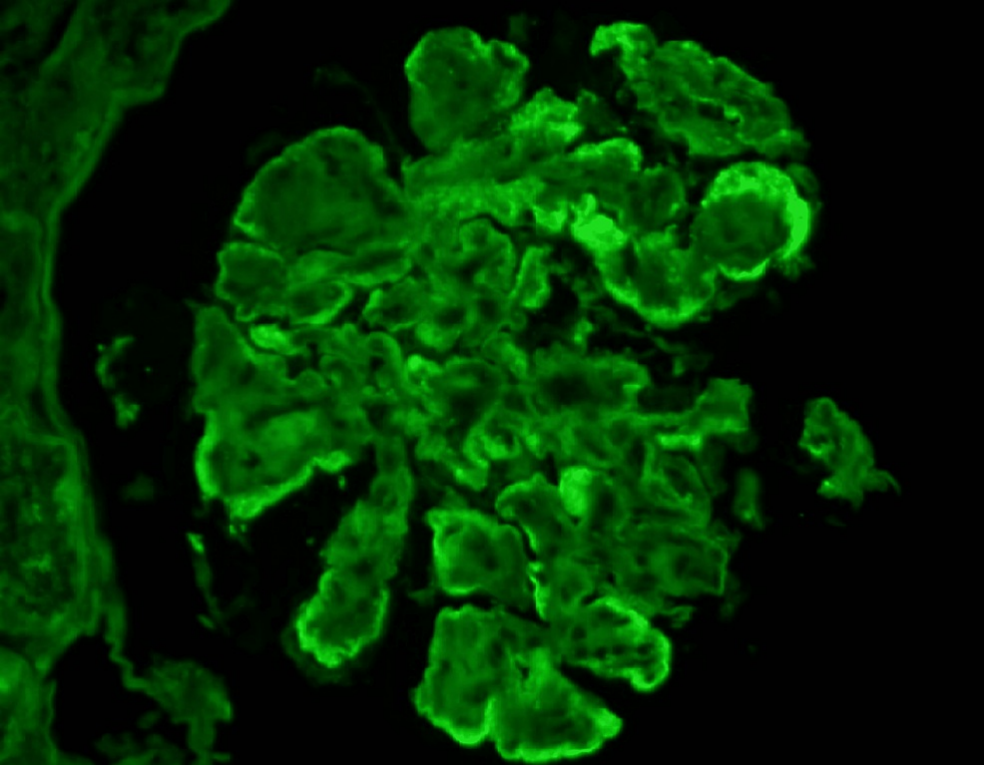
Immunofluorescent microscopy shows segmental mesangial and capillary loop positive staining for IgG

**Figure 3:**
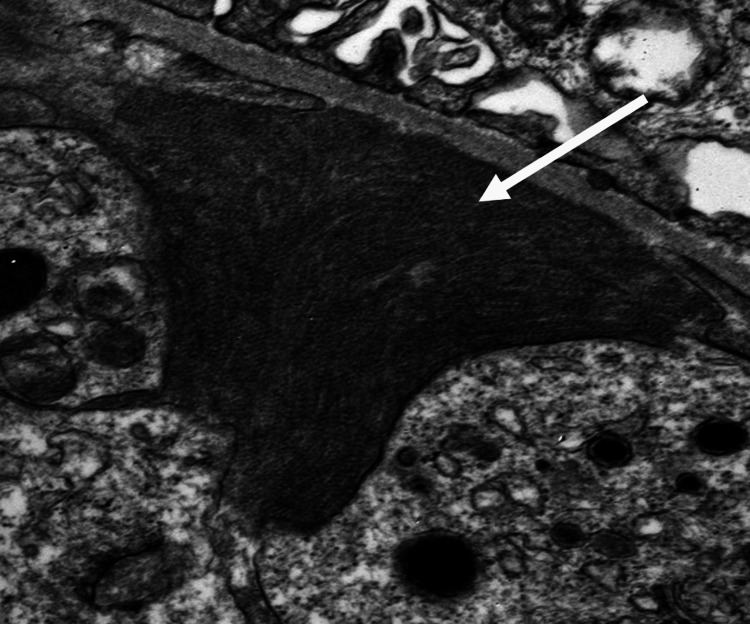
Electron microscopy shows electron-dense deposits in parallel arrays with microtubular substructure (white arrow) (mean diameter 35 nm)

A 74-year-old Caucasian male patient with a history of hypertension, hyperlipidemia, biopsy-proven Immunotactoid GN for which he received six doses of rituximab presents with worsening proteinuria and edema. During the initial diagnosis, he presented with proteinuria of 19 g/24h with negative serologies including hepatitis panel/ANA/DSDNA/SSA/SSB/Cryoglobulins/Complements. His evaluation for malignancy and peripheral flow revealed an abnormal population of B cells concerning for chronic lymphocytic leukemia but bone marrow biopsy, CT scan and PET scan were normal. A renal biopsy was performed, which revealed immunotactoid GN. After initial treatment with rituximab for a total of three doses (days 0, 6 and 14 months), his urine protein creatinine ratio reduced from 19 g/24h to 10.9 g/24h to 1.8 g/24h in six months. He did not receive any other treatment other than rituximab and there were no complications of CLL like hemolytic anemia or hypogammaglobulinemia. His last rituximab dose was one year ago when he came with worsening edema and his proteinuria increased to 3.4 g/24h. Laboratory results showed stable serum creatinine of 1.9 mg/dL with normal protein electrophoresis, serum immunofixation, and slightly elevated kappa and lambda light chains but the ratio was in the normal range. A kidney biopsy was performed for nephrotic range proteinuria and light microscopy showed a membranoproliferative pattern of GN with mild chronicity (Figure 1). Immunofluorescence microscopy (Figure 2) shows segmental mesangial and capillary loop positive staining for IgG. Electron microscopy (Figure 3) shows scattered electron-dense deposits with immunotactoid substructure along the glomerular capillary loops and in the mesangium measuring 59 +/- 6.4 nm in mean diameter. These findings suggest the diagnosis of recurrence of Immunotactoid GN. He was started on rituximab with a reduction of proteinuria from 3.4 g/24h to 1.2 g/24h and was planned to continue every six months. Edema and hypertension are managed with adjusting diuretics and anti-hypertensive medications, respectively. Follow up after one year of starting rituximab, his proteinuria is well controlled (800 mg/24h) with no symptoms.

## Discussion

The glomerular disease may be associated with the appearance of fibrillar deposits in the mesangium or glomerular basement membrane. The fibrils are Congo red positive in amyloidosis and are typically Congo red negative in other forms. The most common forms of nonamyloid glomerular deposition diseases are FG and ITG. Other forms of nonamyloid deposition disease have been described in which the deposits are composed of fibronectin (fibronectin glomerulopathy) or atypical type III collagen fibrils (collagenofibrotic glomerulopathy). Immunotactoid GN is also known as GN with organized monoclonal microtubular immunoglobin deposits and is a very uncommon cause of glomerular disease. In ITG, microtubules are larger than fibrils in FG and amyloidosis (17 to 52 versus 16 to 24 nm versus 10 m in diameter) [3]. The fibrils in FG are randomly arranged, whereas microtubules observed in ITG form parallel bundles [2]. Immunostaining for the marker DNAJB9 is negative in immunotactoid GN which differentiates from Fibrillary GN. Most cases of immunotactoid GN are associated with lymphocytic or plasma cell disorder as some authors call this disorder as GN with organized monoclonal microtubular immunoglobin deposits (GOMMID) [6]. In immunotactoid GN, glomerular deposits are usually composed of monotypic IgG and are usually associated with lymphoproliferative disorders while fibrillary GN had polyclonal immunoglobulin glomerular deposits [6]. Another series mentioned that in immunotactoid GN, hematologic malignancy was identified in 6 out of 16 patients including three with chronic lymphocytic leukemia, two with lymphoma, and two with multiple myeloma. A careful evaluation of B cell or plasma cell lymphoproliferative disease should be part of the evaluation of any patients with immunotactoid GN [7]. This disease is more common in middle-aged persons ranging from 41 to 80 years. Clinical presentation includes proteinuria in all patients with nephrotic syndrome, hematuria in 70%, kidney function impairment in 55%, hypertension in 70% and monoclonal gammopathy in 63%, hypocomplementemia in 33%. Patients with ITG and monoclonal deposits in the kidney had a much higher incidence of hematologic disorders than those with polyclonal deposits. ITG has been described as hepatitis C infection in a minority of patients. Extra renal involvement was seen in a few cases involving bone marrow, skin, and peripheral nerves [6,8,9,10]. 

Diagnosis of ITG is made by kidney biopsy in a patient suspected of glomerular disease. There are no established, noninvasive laboratory tests to diagnose ITG. The serum levels of DNAJB9 were fourfold higher in patients with GN compared with healthy controls and patients with multiple myeloma, immunoglobulin light chain (AL) amyloidosis, and ITG. ITG, in contrast to FG, is characterized by the formation of microtubules on electron microscopy. In addition, these microtubules are typically much larger than the fibrils in FG (17 to 52 versus 16 to 24 nm in diameter). Evaluation for immunotactoid GN includes screening for monoclonal gammopathy with serum and urine electrophoresis with immunofixation and serum-free light chain levels. In those who are confirmed with monoclonal gammopathy, additional investigations including bone marrow examination with flow cytometry, CT scan of the chest, abdomen, and pelvis, and PET scan might be warranted. Immunotactoid GN is very difficult to treat, and no therapies have clearly shown beneficial as there are no randomized controlled trials. Patients with underlying monoclonal gammopathy, malignancy, infection, or autoimmune disease may benefit from treatment of the underlying disorder with B cell or plasma cell targeted therapy. In addition to addressing the underlying disorder, we administer therapies aimed at reducing proteinuria with ACEI/ARBs, controlling blood pressure, and dietary sodium restriction indefinitely with lipid-lowering therapy for hyperlipidemia and weight reduction who are overweight or obese. For those who present rapidly progressive GN and crescents on kidney biopsy, treatment with high-dose glucocorticoids and cyclophosphamide is used in other forms of crescentic GN. Studies have suggested that 50% of patients will have ESRD by five years after diagnosis and the disease may recur after transplantation. Almost 40%-50% of reported patients with ITG develop ESRD within two to six years [11]. The prognosis of immunotactoid GN is comparatively better than fibrillary GN [3,7]. A case series of all patients published from 1977 to 1994 found little if any, difference in clinical outcomes in immunotactoid GN and fibrillary GN [12]. Dialysis or kidney transplantation can be performed in patients with ITG who progress to ESRD. Recurrent disease can occur in the allograft, but the rate of progression is usually slower in the native kidney [13]. A case of spontaneous remission of ITG has been described [14]. Immunosuppressive therapy with glucocorticoids, cyclophosphamide, mycophenolate mofetil, cyclosporin, and azathioprine has been reported in uncontrolled studies with limited and inconsistent results [1]. Rituximab is administered either as four weekly doses of 375 mg/m^2^ intravenously (IV) or as 1 g IV initially followed 14 days later by another 1 g dose, based upon observational data suggesting benefit in some patients [15]. Our case is unique that he responded initially with rituximab to near-complete remission for one year and when the disease recurred, he responded again to rituximab with other conventional treatment measures. Because of the rare occurrence of this disease, more studies are needed to formulate a conventional treatment plan for native and transplant kidney disease.

## Conclusions

Immunotactoid is a rare but distinctly different cause of glomerular disease. This is diagnosed with kidney biopsy by identification of microtubules measuring >30 nm in diameter in electron microscopy. Immunotactoid GN is distinguished from FG by the larger microtubular substructure revealed by electron microscopy (>30 nm compared to ~20 nm). Early referral to a nephrologist will help preserve kidney function as almost 50% patients develop ESRD five years after diagnosis. Serial monitoring of proteinuria and creatinine is important in monitoring the recurrence of Immunotactoid GN.
